# Aberrant Cell Cycle Gene Expression in a Transgenic Mouse Model of Alzheimer’s Disease

**DOI:** 10.3390/cells15020132

**Published:** 2026-01-12

**Authors:** Marika Lanza, Michele Scuruchi, Alessandra Saitta, Rossella Basilotta, Federica Aliquò, Giovanna Casili, Emanuela Esposito, Agata Copani, Salvatore Oddo, Antonella Caccamo

**Affiliations:** 1 Department of Chemical, Biological, Pharmaceutical and Environmental Sciences, University of Messina, Viale Ferdinando Stagno D’Alcontres 31, 98166 Messina, Italy; mlanza@unime.it (M.L.); alessandra.saitta@unime.it (A.S.); antonella.caccamo@unime.it (A.C.); 2 Department of Clinical and Experimental Medicine, University of Messina, Via Consolare Valeria n.1, 98125 Messina, Italy; 3 Department of Chemistry, Biology and Biotechnology, University of Perugia, Via Elce di Sotto, 8, 06123 Perugia, Italy; 4 Department of Biomedical Sciences, Dental Sciences, and Morpho-Functional Imaging, University of Messina, Via Consolare Valeria n.1, 98125 Messina, Italy; 5 Department of Drug and Health Sciences, University of Catania, 95125 Catania, Italy

**Keywords:** Alzheimer’s disease, cell cycle re-entry, differentially expressed genes

## Abstract

Alzheimer’s disease (AD) is increasingly recognized as a disorder that extends beyond amyloid-β (Aβ) and tau pathology. To this end, growing evidence suggests that aberrant neuronal cell cycle re-entry (CCR) may contribute to neurodegeneration. To investigate this mechanism, we profiled the expression of 84 cell cycle-related genes in the brains of aged APP/PS1 mice, a widely used transgenic model of AD, and compared them with age-matched non-transgenic littermates. Our analysis revealed 32 differentially expressed genes (DEGs), 8 of which exhibited significant changes (fold change > 2, *p* < 0.05). Several of these DEGs, including CDC7 and CCNC, displayed consistent dysregulation in human AD brains as assessed using the AMP-AD knowledge portal, supporting their translational relevance. Furthermore, integration with miRNA prediction analyses identified candidate post-transcriptional regulators of these DEGs, highlighting novel layers of regulation. Collectively, our results provide the first systematic overview of cell cycle gene dysregulation in aged APP/PS1 mice, establish cross-species concordance with human AD, and propose miRNA–gene interactions as potential contributors to neuronal vulnerability. These findings underscore the importance of cell cycle pathways in AD pathogenesis and point to new avenues for therapeutic exploration.

## 1. Introduction

Alzheimer’s disease (AD) is the most common cause of dementia worldwide and is characterized by progressive memory loss, cognitive decline, and neuropathological hallmarks including extracellular amyloid-β (Aβ) plaques, intracellular neurofibrillary tangles composed of hyperphosphorylated tau, synaptic dysfunction, and neuronal loss [1]. Although substantial progress has been made in understanding the molecular mechanisms of AD, the precise cascade of events leading to neurodegeneration remains unresolved. Increasing evidence suggests that, in addition to the classical Aβ and tau pathologies, alterations in fundamental cellular processes such as the cell cycle may contribute to disease progression [2].

Post-mitotic neurons are terminally differentiated cells that have permanently exited the cell cycle. Nevertheless, over the past three decades, substantial evidence has accumulated indicating aberrant reactivation of cell cycle-related pathways in neurons vulnerable to AD. Early neuropathological studies reported ectopic expression of cell cycle markers, including cyclins, cyclin-dependent kinases, and DNA replication proteins, in neurons from AD brains but not from age-matched controls [3,4]. A seminal study by Vincent and colleagues demonstrated the presence of cyclins D and E in pyramidal neurons of AD patients, providing the first compelling evidence that neurons in AD attempt to re-enter the cell cycle [5]. Shortly thereafter, Herrup and colleagues showed that neuronal cell cycle re-entry precedes both neurofibrillary tangle formation and neuronal death in human AD brains and in mouse models, positioning aberrant cell cycle activation as an early and potentially causal event in disease pathogenesis rather than a downstream consequence of neurodegeneration [6,7,8]. Subsequent studies reinforced this concept by demonstrating that neurons expressing cell cycle markers can survive for extended periods in a hyperploid or partially replicated state, suggesting that cell cycle re-entry is an abortive process that increases neuronal vulnerability rather than leading to productive division [9,10]. Experimental manipulation of cell cycle regulators further supported a pathogenic role for cell cycle dysregulation: forced cell cycle re-entry promotes neuronal death, whereas inhibition of cyclin-dependent kinases mitigates neurodegeneration and cognitive decline in experimental models [11,12,13]. Collectively, these findings support the hypothesis that aberrant activation of cell cycle machinery is mechanistically linked to neuronal dysfunction and degeneration in AD.

At the same time, the neuronal cell cycle hypothesis has remained controversial. Some studies have failed to detect widespread DNA replication or mitotic progression in AD neurons, leading to the interpretation that cell cycle marker expression may reflect cellular stress responses rather than true cell cycle re-entry [14,15]. Moreover, early bulk transcriptomic analyses of AD brain tissue predominantly highlighted inflammatory, synaptic, and metabolic pathways, with cell cycle-related genes not consistently emerging as dominant signatures [16,17]. These apparent discrepancies likely reflect methodological limitations of earlier approaches, differences in disease stage or brain region analyzed, and dilution of neuron-specific transcriptional signals in heterogeneous tissue samples. Recent advances in transcriptomic technologies, including large-scale RNA sequencing and single-cell and single-nucleus RNA-seq, have renewed interest in the cell cycle hypothesis by enabling more sensitive and cell type-resolved analyses. Single-cell studies have identified distinct neuronal and glial subpopulations in which cell cycle-associated genes are selectively dysregulated in both mouse models and human AD brains [18,19].

The APP/PS1 double transgenic mouse model, which co-expresses human amyloid precursor protein (APP) with the Swedish mutation and presenilin-1 (PS1) with a deletion in exon 9, is widely used to study AD-related amyloid pathology [20]. These mice develop age-dependent Aβ deposition, gliosis, synaptic alterations, and cognitive impairment. Associated with this classical neuropathological phenotype, APP/PS1 mice also develop aberrant cell cycle activity in the brain, making them a suitable model to investigate the role of cell cycle dysregulation in AD pathogenesis [21]. Although individual cell cycle markers have been reported in AD models, a comprehensive analysis of multiple cell cycle-related genes in aged APP/PS1 mice remains lacking. Understanding whether and how cell cycle regulatory networks are altered during the symptomatic stages of AD could shed light on pathogenic mechanisms and identify potential molecular targets for intervention. In this study, we measured the expression levels of 84 cell cycle-related genes in the brains of 18-month-old APP/PS1 mice and age-matched wildtype littermates. Our findings provide a systematic overview of cell cycle gene dysregulation in this widely used AD model and offer insights into the contribution of aberrant cell cycle activity to neurodegeneration.

## 2. Materials and Methods

### 2.1. Animals

Adult male and female B6.Cg-Tg (APPswe, PSEN1dE9)85Dbo/Mmjax mice (30–35 g; 18 months old), hereafter referred to as APP/PS1 mice, along with age- and weight-matched non-transgenic (NonTg) mice with no signs of illness or abnormal behavior at baseline, were housed under standardized conditions. No specific additional inclusion or exclusion criteria were applied. APP/PS1 mice were selected because they represent a well-established model of AD. These mice express a chimeric mouse/human amyloid precursor protein (Mo/HuAPP695swe) and a mutant human presenilin 1 (PS1-dE9), both associated with early-onset familial AD in humans. The humanized APP transgene enables the production of human Aβ peptides, which are central to amyloid plaque formation. This model was chosen because APP/PS1 mice reliably develop age-dependent Aβdeposition in the brain, beginning at 6–7 months of age, thereby recapitulating key neuropathological features of human AD.

All animals were maintained at 22 ± 1 °C with a 12 h light/dark cycle and had free access to food and water. Mice were obtained from The Jackson Laboratory (Stock No. 005864), Bar Harbor, ME, USA. All experimental procedures were conducted in accordance with Italian animal welfare regulations (D.M.116/92), the European Directive (2010/63/EU), and the ARRIVE guidelines. The animal study protocol was approved by the Institutional Animal Care and Use Committee (IACUC) of the Ministero della Salute (protocol code 937/2024; approval date: 27 September 2024).

### 2.2. Immunofluorescence Analysis

Immunofluorescence staining of β-Amyloid 1-42, NeuN, and Ki67 was conducted as previously indicated [22]. Following deparaffinization, the following primary antibodies were incubated overnight on the brain slices: β-Amyloid (1:500; AB5078P Merck, St. Louis, MO, USA), Ki67 (1:100; sc-23900 Santa Cruz Biotechnology, Dallas, TX, USA), and NeuN D4G40 (1:100 24307T Cell Signaling, Danvers, MA, USA). The secondary fluorescent antibodies Alexa Fluor488 goat anti-mouse (A11001; Molecular Probes, Eugene, OR, USA) or anti-rabbit IgG (A11008; Molecular Probes, Eugene, OR, USA) were incubated for three hours the next day, depending on the primary antibody employed. Subsequently, slices were stained with 4′,6′-diamidino-2-phenylindole (DAPI; Hoechst, Frankfurt, Germany). The histological studies were carried out in a blinded manner, and a fluorescence microscope picture (Nikon Eclipse Ci-L microscope, NIKON CORPORATION, Tokyo, Japan) was used to collect images. Images are shown at 20× magnification.

### 2.3. Total RNA Extraction

Total RNA was extracted from mouse cortex and hippocampus according to the manufacturer’s instructions using PureZOL RNA isolation reagent from Biorad (Biorad Laboratories, Inc., Hercule CA, USA). Approximately 100 mg of tissue was homogenized with an Ultra-Turrax tissue homogenizer (IKA, Staufen, Germany) in a glass tube containing 1 mL of ice-cold PureZOL. RNA was then extracted from each sample, and its concentration and purity were assessed using a NanoDrop spectrophotometer (Spectrostar Nano, BMG Labtech, GmbH, Ortenberg, Germany). An A260/A280 ratio between 1.8 and 2.0 was considered indicative of high-quality RNA.

### 2.4. cDNA Synthesis

cDNA was synthesized using the RT^2^ First Strand Kit (Qiagen, GmbH, Hilden, Germany), following the manufacturer’s instructions. Briefly, 2.5 µg of total RNA was treated with the genomic DNA elimination mix before reverse transcription. For each sample, 10 µL of the resulting mixture was used in the reverse transcription reaction.

### 2.5. Real-Time PCR for RT2 Profiler PCR Array

Real-time PCR was performed using a 7500 Real-Time PCR System (Applied Biosystems, Waltham, MA, USA). PCR component mix was obtained using the RT^2^ SYBR^®^ Green qPCR Mastermix (Qiagen, GmbH, Germany). For each reaction, 25 µL of PCR mix was added to the wells of the RT^2^ Profiler PCR Array: Mouse Cell Cycle (Qiagen, GmbH, Germany; Cat. No. 330231). Cycling conditions were set as follows: initial denaturation at 95 °C for 10 min, followed by 40 cycles at 95 °C for 15 s and 60 °C for 1 min. A melting curve analysis was performed at the end of each run to verify PCR specificity.

### 2.6. Western Blot

Animals were euthanized using carbon dioxide, after which the brains were rapidly extracted and divided along the sagittal plane. One hemisphere was immersed in 4% paraformaldehyde for fixation and later immunohistochemical analysis. The remaining hemisphere was snap-frozen and kept at −80 °C until further processing. Frozen hemispheres, with the cerebellum removed, were homogenized in Tissue Protein Extraction Reagent (T-PER; ThermoFisher Scientific, Waltham, MA, USA) supplemented with pepstatin A (0.7 mg/mL), a Complete Mini protease inhibitor cocktail (Roche Applied Science, Penzberg, Germany), and phosphatase inhibitors (Millipore, Burlington, MA, USA). Homogenates were centrifuged and the resulting supernatant was collected as the detergent-soluble fraction.

Equivalent amounts of protein were separated on 10% Bis-Tris SDS–polyacrylamide gels (ThermoFisher Scientific) under reducing conditions and subsequently transferred onto nitrocellulose membranes. Membranes were incubated overnight at 4 °C with the following primary antibodies diluted in 5% milk: cyclin B1 (GNS1), sc-245 (1:200); DP-1 (TFD-10), sc-53642 (1:100); p15^INK4B^/p16^INK4A^ (C-7), sc-377412 (1:100); Cdc7 (DCS-341), sc-56274 (1:100); and recombinant rabbit monoclonal β-actin antibody (0N2H8; HRP-conjugated, Invitrogen, Waltham, MA, USA, MA5-42945; 1:5000). After washing, membranes were incubated for 1 h with the appropriate secondary antibody diluted in 5% milk. Specifically, Goat Anti-Rabbit IgG (H + L) Poly-HRP (Invitrogen 32260; 1:20,000) was used for β-actin, whereas Goat Anti-Mouse IgG (H + L) Poly-HRP (Invitrogen 32230; 1:1000) was used for cyclin B1, DP-1, p15 INK4B/p16 INK4A, and Cdc7.

### 2.7. AMP-AD Knowledge Portal and Data Analysis

Publicly available transcriptomic and metadata from human brain samples were obtained from the Accelerating Medicines Partnership–Alzheimer’s Disease (AMP-AD) Knowledge Portal. The AMP-AD consortium provides harmonized multi-omics datasets generated from post-mortem brain tissue of individuals with Alzheimer’s disease and age-matched cognitively normal controls, including RNA sequencing data from multiple brain regions and independent cohorts. Data were accessed through the AMP-AD Knowledge Portal, which supports standardized processing pipelines, metadata annotation, and cross-cohort analyses. For this study, AMP-AD datasets were queried to assess the expression patterns of the DEGs identified.

### 2.8. Data Analysis and Statistics

CT values were exported into the Excel template provided by the Qiagen GeneGlobe Data Analysis Center (http://www.qiagen.com/geneglobe; accessed on 16 September 2025) and subsequently uploaded to the platform. Samples were grouped into control and test categories. Raw data were normalized to the mean expression of the following housekeeping genes: ACTB (β-actin), GAPDH (glyceraldehyde-3-phosphate dehydrogenase), B2M (β-2-microglobulin), GUSB (β-glucuronidase), and HSP90AB1 (heat shock protein HSP 90-β).

Fold change/regulation was automatically calculated by the platform. Fold regulation provides a biologically meaningful interpretation of fold-change results. Fold-change values greater than 1 indicate positive (up-)regulation, with fold regulation equal to fold change. Fold-change values less than 1 indicate negative (down-)regulation, with fold regulation corresponding to the negative inverse of fold change.

*p*-values were determined using a Student’s *t*-test on replicate 2^−(∆Ct)^ values for each gene in the control and treatment groups. Statistical significance was set at *p* < 0.05.

## 3. Results

**Figure 1:**
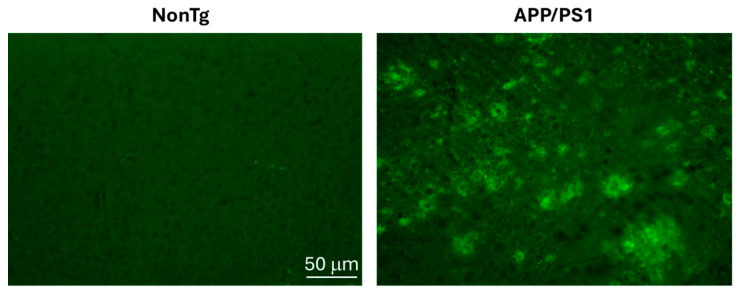
18-month-old APP/PS1 mice have widespread Aβ deposits. Representative cortical sections stained with an Aβ42-specific antibody show Aβ plaques of different sizes spread throughout the cortex of APP/PS1 mice. No plaques were evident in age-matched NonTg mice.

**Figure 2:**
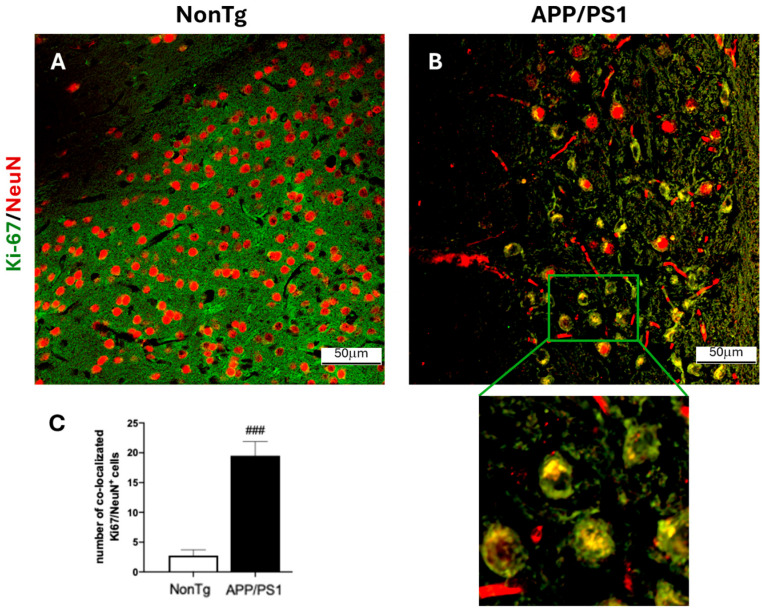
Increased Ki67-positive neurons in the cortex of 18-month-old APP/PS1 mice. (**A**,**B**) Cortical sections from APP/PS1 and age-matched NonTg mice (*n* = 4/genotype) were stained with antibodies against Ki-67 and NeuN. (**C**) Quantification revealed that the number of neurons (NeuN-positive) positive for Ki67 immunoreactivity was approximately four times higher in APP/PS1 mice compared to NonTg controls. Data are expressed as mean ± SD (### *p* < 0.001).

To assess markers of cell cycle re-entry in AD, we employed 18-month-old APP/PS1 mice and age-matched non-transgenic (NonTg) mice (*n* = 4/genotype). At this age, APP/PS1 mice have very well-established Aβ deposits throughout their brain (Figure 1). To investigate whether this robust Aβ deposition is associated with a neuronal attempt to re-enter the cell cycle, we stained cortical sections with Ki-67, a marker of cell proliferation [23]. We found that the number of Ki-67 positive neurons was ~4 times higher in the cortex of APP/PS1 compared to NonTg mice (Figure 2A–C). These results are consistent with the literature (e.g., [24]) and suggest that neurons attempt to re-enter the cell cycle in the brain of 18-month-old APP/PS1 mice.

**Figure 3:**
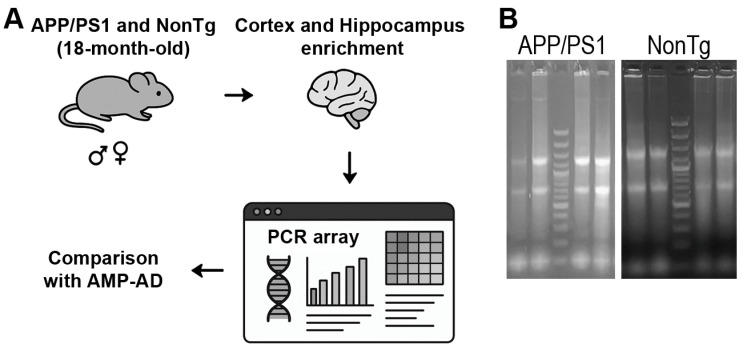
Experimental design and RNA quality control. (**A**) Cartoon schematic of the study design. Cortical and hippocampal enriched tissue was collected from 18-month-old APP/PS1 mice and age-matched NonTg mice. Arrows indicate the workflow from tissue collection and RNA extraction to targeted expression profiling of 84 cell cycle-related genes, differential expression analysis, and comparison with human datasets. (**B**) Representative agarose gel image showing intact 18S and 28S ribosomal RNA bands and minimal degradation, confirming RNA quality.

To further investigate the extent to which cell cycle gene expression is altered in APP/PS1 mice, we extracted total RNA and assessed RNA integrity to ensure suitability for downstream gene expression profiling in 18-month-old APP/PS1 and NonTg mice (*n* = 4/genotype). A schematic overview of the experimental workflow is shown in Figure 3A. After dissection and RNA isolation, we subjected high-quality RNA samples to targeted expression analysis of 84 genes involved in cell cycle regulation. This led to the identification of differentially expressed genes (DEGs) between wild-type and APP/PS1 samples. To evaluate the relevance of our findings to human AD, we further compared the mouse DEG profiles with a publicly available human dataset, AMP-AD. We visualized the extracted RNA on an agarose gel, which showed clear 18S and 28S ribosomal RNA bands (Figure 3B).

**Figure 4:**
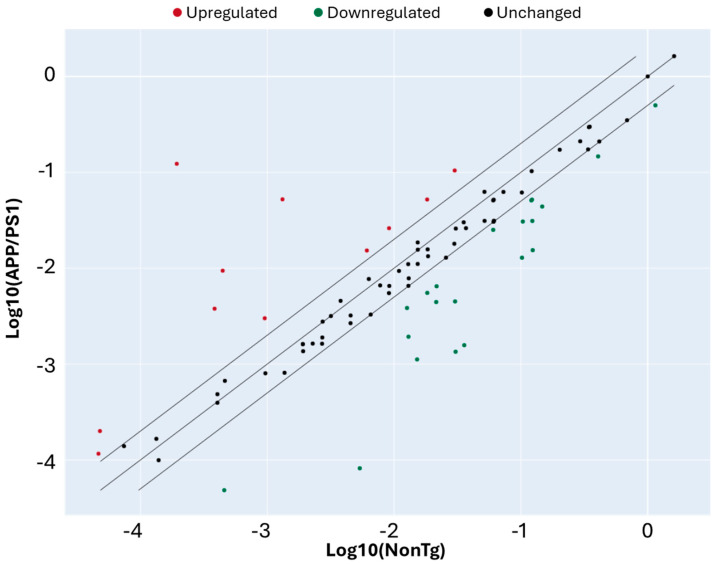
Differential expression analysis of cell cycle-related genes in APP/PS1 and NonTg mice. Scatter plot showing log10-transformed expression levels of 84 cell cycle-related genes in APP/PS1 mice versus NonTg. Each dot represents an individual gene. The central diagonal line indicates equal expression between groups, while the flanking lines mark the twofold change threshold. Genes upregulated in APP/PS1 relative to controls are shown in red, downregulated genes in green, and unchanged genes in black. Several genes deviate beyond the twofold threshold, highlighting transcriptional alterations in the APP/PS1 brain. *n* = 4/genotype.

**Figure 5:**
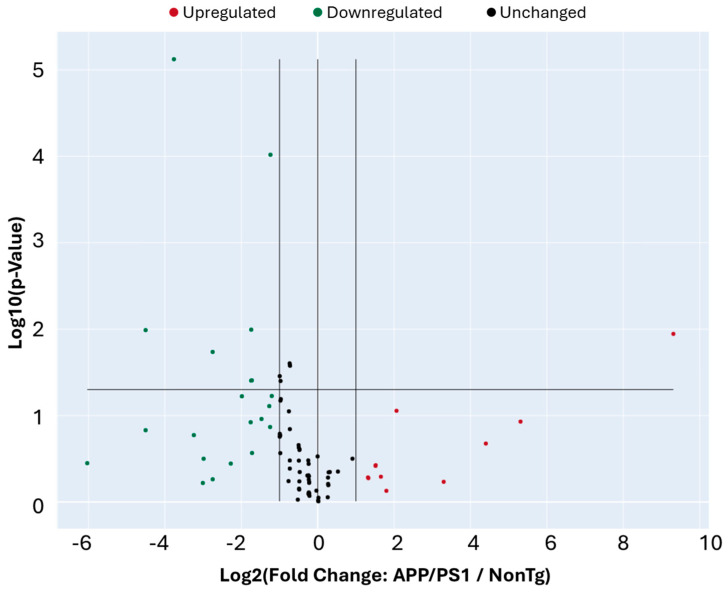
DEGs between APP/PS1 and NonTg. The Volcano Plot identifies significant gene expression changes by plotting the log2 of the fold changes in gene expression on the x-axis versus their statistical significance on the y-axis. The center vertical line indicates unchanged gene expression, while the two outer vertical lines indicate the selected fold regulation threshold. The horizontal line indicates the selected *p*-value threshold. Genes with data points in the far upper left (down-regulated) and far upper right (up-regulated) sections meet the selected fold regulation and *p*-value thresholds. By combining the fold change results with the *p*-value statistical test results, genes with both large and small expression changes that are statistically significant are easily visualized.

We then compared gene expression levels between APP/PS1 and NonTg mice to identify DEGs. Using a twofold change cutoff, we found several genes upregulated (red) and downregulated (green) in APP/PS1 mice relative to NonTg (Figure 4). Most genes clustered along the diagonal, indicating comparable expression between the two groups, while a subset deviated beyond the fold-change thresholds, suggesting significant transcriptional alterations (Table 1). These DEGs represent candidate regulators of aberrant cell cycle activity in the APP/PS1 brain. Statistical analysis indicated that of the 32 genes DEGs that showed a fold change greater than 2, eight also had a *p* < 0.05 (Figure 5, Table 2).

**Figure 6:**
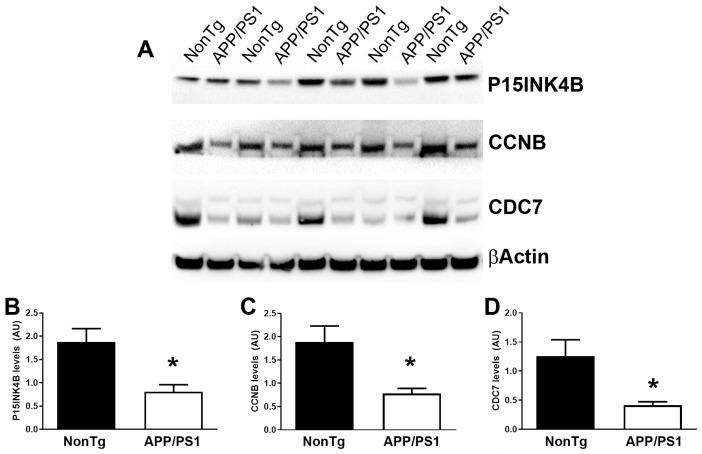
Reduced expression levels of p15-INK4B, CCNB, and CDC7. (**A**) Representative Western blots of proteins extracted from NonTg and APP/PS1 mice (*n* = 5 per genotype). Each blot was probed with the indicated antibodies. β-Actin was used as a loading control. (**B**–**D**) Quantitative analysis of the blots indicated that the levels of p15-INK4B (encoded by the CDKN2B gene), CCNB, and CDC7 were significantly lower in APP/PS1 mice compared to NonTg mice. * *p* < 0.05. AU: arbitrary units.

To determine whether the changes observed at the mRNA level were also reflected at the protein level, we measured the steady-state levels of the products of three randomly selected genes among the eight DEGs with *p* < 0.05 (listed in Table 2). We found that the protein levels of p15-INK4B (encoded by CDKN2B), CCNB, and CDC7 were significantly reduced in APP/PS1 mice compared to age-matched NonTg mice (Figure 6).

**Figure 7:**
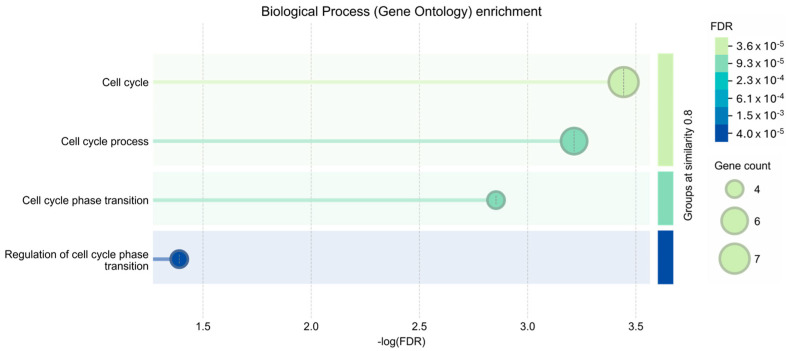
Gene Ontology (GO) enrichment analysis of biological processes. The bar plot displays the most significant biological process terms enriched in the DEGs. The terms are plotted against the negative logarithm of the False Discovery Rate (−log(FDR)) on the x-axis, indicating statistical significance. The size of each dot corresponds to the number of genes associated with the GO term (Gene count), while the color gradient represents the FDR value. Terms are clustered based on semantic similarity (threshold = 0.8), highlighting a predominant enrichment in cell cycle-related pathways.

To gain insight into the biological functions of the eight DEGs with *p* < 0.05 (listed in Table 2), we performed Gene Ontology (GO) over-representation analysis. As expected, these genes were primarily associated with the cell cycle, including the regulation of cell cycle transitions (Figure 7).

**Figure 8:**
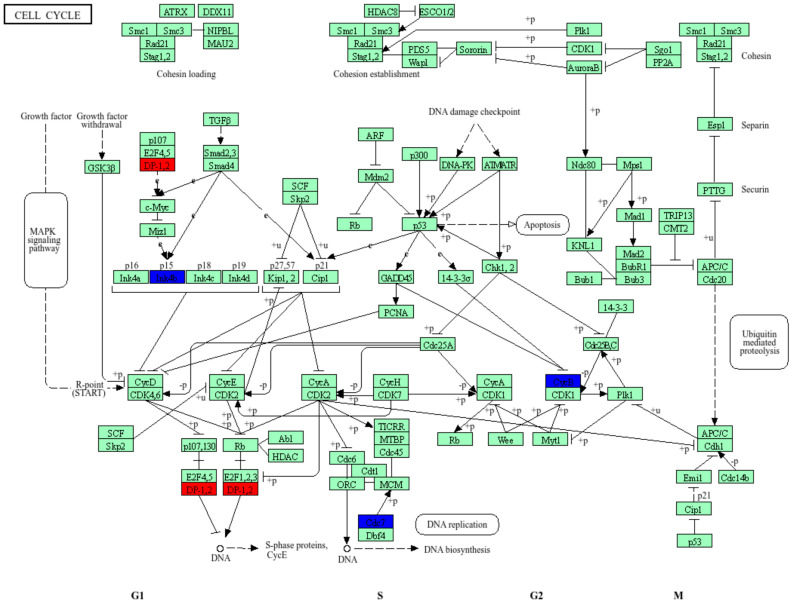
Pathway enrichment analysis. Using the KEGG database (map04110, Cell Cycle; Kanehisa Laboratories), we performed a pathway enrichment analysis to highlight the main regulatory networks controlling eukaryotic cell cycle progression through the G1, S, G2, and M phases. Upregulated genes are shown in red, and downregulated genes are shown in blue.

We further examined the cellular localization and pathway involvement of these genes using the KEGG pathway database. As shown in Figure 8, several of the DEGs identified in our dataset, including Cdc7, CcnB2, Cdkn2b, Ccnc, Tfdp1, Ppm1d, and Rad51, mapped to key checkpoints that govern DNA replication, damage repair, and mitotic progression. Specifically, Cdc7 is essential for the initiation of DNA replication at the G1/S transition; CcnB2 and Ccnc regulate CDK activity during the S and G2/M phases, while Cdkn2b acts as a CDK inhibitor mediating p53-dependent cell cycle arrest under genotoxic stress. Rad51 contributes to homologous recombination and double-strand break repair, whereas Ppm1d (Wip1) negatively modulates the DNA damage response by dephosphorylating p53 and ATM/ATR substrates. The coordinated alteration of these genes suggests dysregulation of cell cycle control and DNA damage checkpoint signaling, potentially contributing to neuronal cell cycle re-entry and genomic instability observed in AD models.

To further explore potential post-transcriptional regulation, we generated a table of predicted and validated mouse microRNAs (miRNAs) targeting our DEGs (Table 3). Our analysis identified several candidates, including miR125b, predicted to target CCNC, and miR-29b, associated with CDC7. Integrating these findings with AMP-AD data highlights miRNAs that may regulate disease-relevant gene expression changes in both mouse and human brains, providing a prioritized list for future experimental analysis.

To evaluate the relevance of our findings in human AD pathology, we leveraged the Accelerating Medicines Partnership–Alzheimer’s Disease (AMP-AD) knowledge portal. AMP-AD is a large-scale integrative resource that provides multi-omic data, including transcriptomic, proteomic, and genomic profiles from multiple brain regions of well-characterized human cohorts [45]. Importantly, AMP-AD also includes network-based analyses that facilitate the identification of gene-gene interactions and modules associated with disease traits, allowing for cross-species comparisons of experimental findings.

Using this platform, we queried the expression patterns of the eight DEGs identified in our mouse model across multiple human brain regions. Several DEGs showed consistent dysregulation in AMP-AD datasets, suggesting translational relevance (Table 3). For example, CDC7 and CCNC were also significantly downregulated in the anterior cingulate cortex (ACC), Inferior frontal gyrus (IGF), parahippocampal gyrus (PHG), Superior Temporal Gyrus (STG), and temporal cortex (TCX). CCNC was also significantly downregulated in the dorsolateral prefrontal cortex (DLPFC). Overall, these results provide robust cross-species comparisons for our identified DEGs and underscore the utility of AMP-AD networks in bridging preclinical findings with human AD pathophysiology (Table 4).

Collectively, these results demonstrate that the DEGs identified in our mouse model are not only conserved in human AD but may also be subject to specific miRNA-mediated regulation, underscoring their potential therapeutic relevance.

## 4. Discussion and Conclusions

The present study provides a comprehensive survey of cell cycle-related gene expression in the brains of aged APP/PS1 mice, uncovering transcriptional signatures that align with the hypothesis of aberrant neuronal cell cycle re-entry in AD. While previous studies have reported individual markers of cell cycle activity in human AD and mouse models [5,6,21], our systematic approach identifies a broader set of dysregulated genes, several of which exhibit conserved alterations in human brains.

Neurons are terminally differentiated, but accumulating evidence shows that they can aberrantly express cell cycle proteins under pathological conditions [13]. Importantly, cell cycle re-entry precedes tau pathology and neuronal death, suggesting it is not a secondary byproduct but rather a potential initiating factor in neurodegeneration [6]. Our findings reinforce this view by revealing the molecular signature of abortive cell cycle re-entry in neurons. On one hand, the marked increase in Shc1 transcripts may reflect hyperactivation of Shc1-mediated signaling to proliferative kinase pathways [46], accompanied by the downregulation of a key brake on G1 progression, Cdkn2b [47]. On the other hand, the downregulation of transcripts encoding critical components of the DNA replication machinery, such as Cdc7 and Tfdp1, which are required for replication origin firing [48] and transcription of genes necessary for S-phase progression [49], respectively, suggests that progression through S phase is stalled. Consistently, the reduced expression of the late-stage cyclin Cyclin B2 indicates that neurons fail to enter M phase [50].

In this context, neurons appear to have a diminished capacity to cope with DNA damage. With an active p53-dependent DNA damage checkpoint, maintained through Ppm1d downregulation [51] and combined with reduced Rad51 expression, which normally mediates the repair of replication-associated DNA damage [52], the process becomes essentially suicidal for the neuron. Finally, the downregulation of Ccnc [53] may be particularly significant, as it could simultaneously facilitate cell cycle progression by removing transcriptional repression of cell cycle genes and blocking the neuron’s apoptotic escape route, resulting in a pathological state of lock-in.

A caveat to our data is that they are derived from bulk tissue RNA profiling. Consequently, the resulting transcriptomic signals represent an average across all brain cell types, potentially obscuring cell-type-specific responses, particularly the distinct contributions of neurons and glia. Notably, although reactive glial cells in APP/PS1 mice can proliferate, their transcriptomic signature would be expected to show widespread upregulation of proliferation-related genes, including cyclins. Instead, the dominant pattern observed in our data is a downregulation of these genes, strongly suggesting that the neuronal signature underlies this specific transcriptional profile.

One limitation of many transgenic AD models is the incomplete overlap with human pathology. By integrating our results with AMP-AD datasets, we provide evidence that at least a subset of the dysregulated genes in APP/PS1 mice are conserved in human AD brains. Importantly, because AMP-AD datasets are derived from bulk RNA sequencing of post-mortem human brain tissue, the overlap observed between mouse and human datasets directly implies that some of the transcriptional alterations identified here are detectable in human AD. These cross-species comparisons strengthen the utility of the APP/PS1 model for investigating cell cycle dysregulation, while also identifying candidate genes for future mechanistic studies. Notably, for the mouse studies, total RNA was extracted from the cortex and hippocampus of aged APP/PS1 mice and age-matched NonTg littermates. These brain regions were selected because they are critically involved in AD pathology and are commonly used for assessing neurodegenerative and synaptic alterations in this model. For the human analyses, gene expression data were obtained from publicly available datasets accessed through the AMP-AD Knowledge Portal. The specific human brain regions included in the analyses are detailed in Table 4. These datasets comprise transcriptomic profiles from multiple AD-relevant cortical regions. Although an exact one-to-one regional correspondence between mouse and human samples is not feasible due to differences in dataset availability, the analyzed human regions include cortical areas that are biologically comparable to the mouse cortex and are prominently affected in AD. Moreover, this conservation across species suggests that cell cycle-related transcriptional changes, while potentially subtle and diluted in whole-tissue analyses, represent a conserved molecular feature of AD rather than a model-specific artifact, and may be further resolved at higher cellular resolution by single-cell transcriptomic approaches.

Our integration of miRNA predictions suggests that specific miRNAs may contribute to the observed gene expression changes. This aligns with growing evidence that post-transcriptional regulation is a critical layer of AD pathophysiology [54]. Several miRNAs have been implicated in both Aβ metabolism and neuronal survival, and our results extend this role to the regulation of cell cycle genes. Experimental validation of these interactions will be essential to determine whether targeting miRNAs could restore proper cell cycle control in vulnerable neurons. To this end, the identified miRNA–gene relationships are predictive and intended to guide future mechanistic studies.

Growing evidence highlights a partial convergence in AD with molecular pathways classically associated with cancer biology. Among these, dysregulation of cell cycle control and p53-dependent signaling has received increasing attention. In proliferative cells, activation of cell cycle machinery promotes division and survival; however, in post-mitotic neurons, aberrant cell cycle re-entry is widely viewed as a maladaptive response that precedes neuronal dysfunction and death. Consistent with this concept, our analysis revealed altered expression of multiple cell cycle-related genes in aged APP/PS1 brains, several of which have well-established roles in DNA replication and transcriptional control in dividing cells. Notably, our miRNA prediction analyses identified several candidate regulatory miRNAs, such as the miR-34 family, miR-15a/16, and miR-125b, which are canonical components of tumor suppressor networks and are tightly linked to p53 signaling. In cancer, these miRNAs act to restrain cell cycle progression and promote apoptosis in response to genomic stress, whereas in neurons their dysregulation has been associated with aberrant cell cycle reentry, synaptic dysfunction, and neurodegeneration. The identification of these shared regulatory nodes suggests that neurons in the AD brain may engage conserved stress-response programs that are also operative in oncogenesis, but with fundamentally different cellular outcomes. Together, these findings support the notion that AD and cancer, despite their divergent clinical manifestations, share overlapping molecular mechanisms centered on cell cycle control and stress signaling pathways. Importantly, our results extend this concept by providing a systematic overview of cell cycle gene dysregulation in an AD mouse model and by implicating miRNA-mediated regulation as a potential contributor to these cancer-like signaling programs in vulnerable neurons.

Aberrant activation of cell cycle pathways presents a potential therapeutic target. Pharmacological inhibition of cell cycle kinases has shown promise in preclinical studies [2], but concerns remain regarding specificity and safety. Our dataset provides a refined set of candidate genes whose modulation may selectively impact pathological processes in neurons without broadly impairing cell division in proliferating tissues.

The use of bulk brain tissue precludes cell-type-specific resolution. Thus, future studies are needed to leverage single-cell transcriptomics to identify whether dysregulated genes are enriched in neurons versus glia, and whether their expression correlates with Aβ deposition or tau pathology. Additionally, longitudinal studies in younger APP/PS1 mice could determine whether these alterations emerge prior to overt amyloid deposition, providing further evidence of causality.

Overall, our study establishes a systematic map of cell cycle gene dysregulation in aged APP/PS1 mice, demonstrates conservation with human AD brains, and implicates miRNAs as potential regulators of these changes.

Together, these findings support the hypothesis that aberrant cell cycle activation contributes to AD pathogenesis and opens new avenues for mechanistic and therapeutic exploration.

## Figures and Tables

**Table 1:** Differentially Expressed Genes in APP/PS1 vs. NonTg Mice.

Gene Name	Gene Symbol	Fold Regulation
Transcription factor Dp-1	Tfdp1	634.56
MutS Homolog 2	Msh2	39.64
Stratifin (14-3-3 sigma)	Sfn	21.13
Cyclin D3	Ccnd3	9.83
Tumor protein p63	Trp63	4.18
Structural maintenance of chromosomes 1A	Smc1a	3.47
E2F transcription factor 1	E2f1	3.15
Growth arrest and DNA damage-inducible alpha	Gadd45a	2.86
Glucuronidase beta	Gusb	2.86
Heat shock protein 90 alpha family class B member 1	Hsp90ab1	2.51
RAD9 checkpoint clamp component A	Rad9a	2.49
RAS-related nuclear protein	Ran	−2.30
Protein phosphatase, Mg^2+^/Mn^2+^-dependent 1D	Ppm1d	−2.36
Polycystin 1	Pkd1	−2.37
ATM serine/threonine kinase	Atm	−2.41
Cyclin D2	Ccnd2	−2.77
BCL2 apoptosis regulator	Bcl2	−3.29
Cyclin-dependent kinase inhibitor 2B	Cdkn2b	−3.30
RAD51 recombinase	Rad51	−3.34
Cell division cycle 7-related protein kinase	Cdc7	−3.35
Mitotic arrest deficient-like 1	Mad2l1	−3.38
HUS1 checkpoint clamp component	Hus1	−3.96
Glyceraldehyde-3-phosphate dehydrogenase	Gapdh	−4.85
Cyclin B2	Ccnb2	−6.73
Cyclin E1	Ccne1	−6.73
DNA damage inducible transcript 3 (CHOP)	Ddit3	−7.93
Cyclin-dependent kinase 4	Cdk4	−8.04
Checkpoint kinase 2	Chek2	−9.47
SHC adaptor protein 1	Shc1	−13.59
Cyclin C	Ccnc	−22.72
Cyclin-dependent kinase regulatory subunit 1B	Cks1b	−22.73
Beta-actin	Actb	−65.53

**Table 2:** List of selected genes with significant dysregulation. Genes are shown with their full names and ranked according to *p*-value (ascending order).

Gene Symbol	Gene Name	*p*-Value
Shc1	SHC Adaptor Protein 1	0.000008
Ppm1d	Protein Phosphatase, Mg^2+^/Mn^2+^-Dependent 1D	0.000096
Rad51	RAD51 Recombinase	0.010135
Ccnc	Cyclin C	0.010261
Tfdp1	Transcription Factor Dp-1	0.011345
Ccnb2	Cyclin B2	0.018341
Cdkn2b	Cyclin-Dependent Kinase Inhibitor 2B	0.039137
Cdc7	Cell Division Cycle 7	0.039379

**Table 3:** Predicted and validated microRNAs (miRNAs) targeting our DEGs. The miRNA listed in bold are expressed in the brain.

Genes	miRNAs	References
RAD51	**miR-103, miR-107, miR-155, miR-182, miR-96, miR-34a/b/c, miR-214-5p**	[25,26,27,28,29,30]
CDC7	**miR-29b-3p**	[31]
CDKN2B	**miR-15a-5p, miR-429**	[32,33]
CCNB2	**miR-335-5p, miR-205**	[34,35]
TFDP1	miR-4711-5p	[36]
SHC1	**miR-452, miR-365;** miR-5582-5p	[37,38,39]
PPM1D (WIP1)	**miR-129-1-3p, miR-16-5p, miR-29 family**	[40,41,42]
CCNC (Cyclin C)	**miR-125b; miR-206**	[43,44]

**Table 4:** Dysregulated genes in AMP-AD datasets. Analysis of significance values (*p*-values) for the differential expression of cell cycle-related genes across different human brain regions. Highlighted yellow boxes indicate statistically significant associations (*p* < 0.05), suggesting a potential region-specific regulation of these genes in the context of neurodegenerative processes. The risk score represents a composite index derived from the weighted expression of these candidate genes, reflecting the overall contribution of their dysregulation to disease susceptibility. Abbreviations: Anterior cingulate cortex (ACC), cerebellum (CBE), dorsolateral prefrontal cortex (DLPFC), frontal pole (FP), inferior frontal gyrus (IGF), posterior cingulate cortex (PCC), parahippocampal gyrus (PHG), Superior Temporal Gyrus (STG), temporal cortex (TCX).

		CDC7	PPM1D	CCNC	SHC1	CDKN2B-AS1	TFDP1	RAD51	CCNB2
**RISK SCORE**		3.58	2.53	2.42	1.19	0.81	0.55	1.38	0.89
**ACC**	**L2FC**	−0.117	0.132	−0.0922	−0.106	−0.0729	0.11	-	-
	***p*-VALUE**	0.0433	0.000145	0.0045	0.147	0.467	0.000279	-	-
**CBE**	**L2FC**	−0.0341	0.194	−0.0913	0.000867	-	0.0118	0.265	-
	***p*-VALUE**	0.656	0.00116	0.0836	0.992	-	0.843	0.000975	-
**DLPFC**	**L2FC**	−0.0821	0.194	−0.0644	−0.2	−0.0346	0.0616	-	-
	***p*-VALUE**	0.0661	0.00116	0.0177	0.000217	0.721	0.0201	-	-
**FP**	**L2FC**	−0.175	0.113	−0.0248	−0.0164	0.073	0.00775	−0.251	−0.345
	***p*-VALUE**	0.053	0.0338	0.621	0.889	0.491	0.88	0.033	0.0104
**IGF**	**L2FC**	−0.248	0.131	−0.0926	0.0955	0.0793	0.00603	−0.103	−0.925
	***p*-VALUE**	0.00262	0.00709	0.0311	0.302	0.476	0.904	0.451	0.545
**PCC**	**L2FC**	−0.121	0.172	−0.0854	−0.121	−0.1	0.0597	-	-
	***p*-VALUE**	0.0279	0.0000051	0.0119	0.105	0.314	0.0777	-	-
**PHG**	**L2FC**	−0.354	0.285	−0.0806	0.17	0.325	−0.0923	0.196	0.148
	***p*-VALUE**	0.00000159	6.35 × 10^−11^	0.0335	0.0297	0.0000537	0.00708	0.0639	0.216
**STG**	**L2FC**	−0.27	0.138	−0.122	0.13	0.06	−0.0527	−0.0922	−0.1
	***p*-VALUE**	0.00111	0.00512	0.00443	0.157	0.603	0.211	0.511	0.513
**TCX**	**L2FC**	−0.236	0.342	−0.15	0.299	-	0.0801	0.097	-
	***p*-VALUE**	0.00153	6.69 × 10^−7^	0.00387	0.0000149	-	0.0865	0.343	-

## Data Availability

The original contributions presented in this study are included in the article. Further inquiries can be directed to the corresponding author.

## Abbreviations

The following abbreviations are used in this manuscript:

AD	Alzheimer’s disease
Aβ	Amyloid-β
APP	Amyloid precursor protein
PS1	Presenilin 1
PS2	Presenilin 2
DEGs	differentially expressed genes
CCR	Cell cycle re-entry
ACTB	β-actin
GAPDH	glyceraldehyde-3-phosphate dehydrogenase
B2M	β-2-microglobulin
GUSB	β-glucuronidase
HSP90AB1	heat shock protein HSP 90-β
NonTg	non-transgenic
AMP-AD	Accelerating Medicines Partnership–Alzheimer’s Disease
